# Alcohol’s contribution to climate change and other environmental degradation: a call for research

**DOI:** 10.1093/heapro/daae004

**Published:** 2024-02-02

**Authors:** Megan Cook, Nathan Critchlow, Rachel O’Donnell, Sarah MacLean

**Affiliations:** Institute for Social Marketing and Health, Faculty of Health Sciences and Sport, University of Stirling, Stirling, FK9 4LA, Scotland, UK; Centre for Alcohol Policy Research, School of Psychology and Public Health, La Trobe University, Plenty Road, Kingsbury Dr, Bundoora VIC 3086, Australia; Institute for Social Marketing and Health, Faculty of Health Sciences and Sport, University of Stirling, Stirling, FK9 4LA, Scotland, UK; Institute for Social Marketing and Health, Faculty of Health Sciences and Sport, University of Stirling, Stirling, FK9 4LA, Scotland, UK; Centre for Alcohol Policy Research, School of Psychology and Public Health, La Trobe University, Plenty Road, Kingsbury Dr, Bundoora VIC 3086, Australia; School of Allied Health, Human Services & Sport, La Trobe University, Plenty Road, Kingsbury Dr, Bundoora VIC 3086, Australia

**Keywords:** alcohol, climate change, public health, environment, fast-moving consumer goods

## Abstract

Climate change is the single biggest health threat facing humanity. The production, distribution and consumption of many fast-moving consumer goods contribute substantially to climate change, principally through releasing greenhouse gas emissions. Here we consider just some of the ways that alcohol—already a key contributor to an array of health, social and economic burdens—exacerbates environmental harms and climate change. We explore current evidence on alcohol production as a resource- and energy-intensive process, contributing to significant environmental degradation through water usage and other carbon emission costs. We argue that the impacts of alcohol production on climate change have been minimally explored by researchers. Yet the extent of the unfolding catastrophe beholds us to consider all available ways to mitigate unnecessary emissions, including from products such as alcohol. We then turn to suggestions for a research agenda on this topic, including investigations of commercial determinants, inequalities and product advice to help consumers choose lower-carbon options. We conclude by arguing that public health researchers already have an array of methodological expertise and experience that is well placed to produce the evidence needed to inform regulation and efforts by alcohol producers and consumers to minimize their contributions to environmental harms.

**Contribution to Health Promotion**

- Climate change is projected to have escalating catastrophic effects on ecosystems and humans.
- Alcohol—already a key contributor to an array of health, social and economic burdens—exacerbates climate change in several ways, starting with production right through storage and disposal of containers and consumption itself.
- The extent of the unfolding climate catastrophe beholds us all to consider ways to mitigate unnecessary emissions, including from products such as alcohol.

## INTRODUCTION

### Climate crisis as a public health issue

The greatest threat to global public health is the continued failure of world leaders to keep the global temperature rise below 1.5°C and to restore nature. (Atwoli *et al.*, 2021)

Climate change is projected to have escalating catastrophic effects on ecosystems and humans, being the single greatest health threat facing humanity (World Health Organization [WHO], 2021; IPCC, 2023). Between 2030 and 2050, the WHO estimates that climate change will cause ~250 000 additional deaths per annum, from factors such as malnutrition, malaria, diarrhoea and heat stress (WHO, 2021). Already, increasingly frequent extreme weather events are spreading diseases (McDermott, 2022), disrupting food systems (Richards *et al*., 2021), contributing to water insecurity (Betts *et al*., 2018) and mental health issues (Aylward *et al*., 2022). The WHO estimates that climate change will directly cost health (including health outcomes, facilities and systems) from 2 to 4 billion USD annually by 2030 (WHO, 2021).

The production, distribution and consumption of many of our fast-moving consumer goods contribute substantially to climate change, principally through releasing greenhouse gas (GHG) emissions. While there is growing evidence on the carbon and environmental costs of food products, such as meat and dairy (Ritchie and Roser, 2023), there is little commensurate knowledge about alcohol products. Such insight is needed to inform industry regulation and consumer choice around whether to drink alcohol and, if they do, how much and which products they choose to minimize degradation. In this piece, we begin by considering just some of the ways in which alcohol—already a key contributor to an array of health, social and economic burdens (Babor, 2022)—exacerbates climate change. We then make a case for future academic research on this topic, arguing that further evidence is needed on alcohol’s impact on environment and the climate.

### Climate impacts of alcohol

Alcohol production is an energy-intensive process, from farming through to bottling, requiring significant quantities of water—taking around 800 l of water to produce 1 l of wine (IAS, 2022). Wine production generates large quantities of wastewater contaminated with organic material from grapes, cleaning and disinfection products, and products used for wine treatment (Conradie *et al.*, 2014). This causes significant harm, further polluting soils and waterways. For example, reports of waste from alcohol production being dumped into the Waki River in Uganda caused fish to die and impacted communities’ drinking water sources (Kasamba and Mugo, 2007), distilleries have been fined for polluting local waterways with manufacturing waste (Adie, 2023), and assessments of Australian wineries reported poor wastewater quality and toxic effects on local ecosystems (Kumar *et al.*, 2006). Put in such stark terms, we find ourselves in a situation where contaminated water sources are a major contributor to illness and mortality. Between 2000 and 2017, an estimated 2.2 billion people did not have access to safe drinking water (WHO, 2019), and many countries are facing extreme droughts (United Nations, 2021). Yet clean drinking water is being used to produce a consumer commodity with vast social, economic and health costs. For example, in Australia, the social, economic and health costs were conservatively estimated at $66.8 billion in 2017/2018 (Whetton *et al*., 2021).

Although water usage is a significant concern related to production, the cost in carbon emissions is perhaps even greater. Estimates suggest that the production of 1 l of beer produces between 510 and 842 g of CO_2_ [although this depends on packaging (Amienyo and Azapagic, 2016)]. Estimates from Sweden indicate that, on average, the production of wine and other liquors generates three times higher GHG emissions per litre compared to beer, albeit the degree of climate harm from different stages of the production processes varies for different types of alcohol products (Hallström *et al*., 2018). Considering the environmental impact of alcohol in relation to different discretionary food groups, an Australian estimate found that while processed meats (15%) contribute most substantially to CO_2e_, this was followed by condiments and confectionary (5%) and alcohol (5%) (Hadjikakou, 2017). In the United Kingdom, alcohol accounts for 1.46% of the UK’s total GHG emissions, with nearly 0.6% coming from consumption of alcohol (primarily related to drinking in on-premises settings) (Garnett, 2007).

While there have been significant advancements in alcohol packaging (Carruthers, 2021) and most glass bottles are recyclable, packaging continues to generate substantial waste. In the United Kingdom, for example, an estimated 50% of alcohol containers that could be recycled are not (Myers, 2019; IAS, 2022). The production of packaging also produces substantial CO_2_ emissions, with beer can emissions estimated at over 340 000 tonnes annually in the United Kingdom [contributing 0.052% to the UK’s total GHG emissions (Garnett, 2007)]. These carbon costs can often be modified. For example, packaging wine in lighter glass bottles, using more recycled glass content than single-use glass, or shipping wines in vats and bottling them locally would reduce emissions (Amienyo *et al*., 2015; Morgan *et al*., 2022).

No and low-alcoholic beverages are also the result of energy intensive dealcoholization—‘a process in which the alcohol produced during the fermentation is removed from the beer by different methods in accordance with the standards of low-/free-alcohol beer’ (Sohrabvandi *et al*., 2010)—although the exact climate damage has not been estimated in any studies we are aware of. The carbon costs of homebrewing are similarly (to the best of our knowledge) unknown. Finally, the environmental costs of transportation, even locally within countries, are particularly a concern, and alcohol is a major export for many nations, including Australia, which in 2022 exported 625 327 000 l of wine (Morgan *et al*., 2022; Wine Australia, n.d.).

Thus, the factors that must be calculated in assessing the carbon cost of alcohol are extensive, including those entailed in replacing woodlands with alcohol-producing plants, building and maintaining production and storage machinery and units, fertilizers, pesticides and other chemicals required in production, producing packaging and labels, electricity, fuels, other transport costs and waste management (Carballo Penela *et al*., 2015). Using a comprehensive method, Carballo Penela *et al*. calculate the carbon footprint of a 75 cl bottle of wine from a small Spanish vineyard at 3817 g of CO_2_. However, there is little consensus on how to measure the environmental impacts of beverage production, and thus estimates vary widely depending on the measures used and factors considered.

While further scoping is clearly needed to understand the true environmental costs and how they vary for different alcoholic products, the effects are manifold, starting with production and extending to storage, disposal of containers and consumption itself. The impacts of alcohol product production on climate change have been minimally explored by alcohol researchers. Yet the extent of the unfolding catastrophe beholds us all to consider ways to mitigate unnecessary emissions, including from products such as alcohol that hold a central place in domestic life (MacLean *et al*., 2022). We now turn to some broader considerations that might underpin a research agenda on this topic.

### Commercial determinants—industry greenwashing

In recent years, increasing attention has been paid to the ways that commercial sector activities impact public health—the commercial determinants of health—including in relation to non-communicable diseases, such as alcohol consumption, smoking, diet and gambling. Powerful transnational corporations and commercial interests pursue profits at the expense of existing economic, social and ethnic inequalities, with little accountability. These corporations influence and exploit weaker regulations and enforcement standards in low- and middle-income countries, exacerbating inequalities in non-communicable diseases and environmental damage from unhealthy produce use (Gilmore *et al*. 2023). As such, commercial interests present a significant threat to public health and planetary health (LSHTM and Petticrew, 2022; Freudenberg *et al*., 2023). However, the wider impacts of these commercial interests, including indirect harms on the environment, are rarely considered in relation to alcohol. This needs to be part of the broader conversation to address the current power imbalance, which has arguably led to a state of policy inertia when it comes to the climate crisis (Ciplet *et al.*, 2015). Examining, deconstructing and understanding the impact of industry greenwashing on the public and policymakers is an important potential starting point for broadening the current dialogue.

Greenwashing has been defined as ‘deliberate corporate action with the presence of misleading elements, focussed on the deception of stakeholders’ (de Freitas Netto *et al*., 2020). Central to contemporary greenwashing is the language of win–win solutions. This language shifts the focus of debates from moral responsibility to practical and technical solutions, while at the same time failing to offer any viable response to environmental problems and concerns (Vollero, 2022). Take, for example, Heineken’s ‘Brew a Better World’ report, which states:

Our strategy is based on the four Rs: Reduce, Replace, Remove and Report. We are working to decrease absolute carbon emissions across the entire value chain – from barley to bar – with supplier engagement and sustainable sourcing playing an important role. (Heineken, n.d.)

To the best of our knowledge, no systematic research has focused specifically on alcohol industries’ greenwashing practices. However, many alcohol companies report, and even advertise, their corporate social responsibility activities, in which the sustainable development goals (SDGs) and their actions related to the environment feature prominently. For example, in an exploratory review of varying corporate social responsibility agendas disseminated by spirit producers (i.e. not just focused on the environment), Jones, Hillier and Comfort find that ‘environmental issues loom large’, with companies reporting on their commitment to water conservation and achievements in improving energy efficiency—‘William Grant and Sons claims to be “*at the forefront of best practice regarding energy efficiency in the manufacture of distilled spirits*”’ [(Jones et al., 2013), p. 7]. This remains an important area for future research; knowledge of greenwashing will enable better regulatory choices and help researchers understand how industry claims stack up against independent research.

When considering greenwashing, practices of green marketing, which broadly refer to the promotion or advertising of products with eco-concerns (Sarkar, 2012), also need to be examined. Green marketing is often used as a tool for demonstrating sustainable development and, at the same time, strengthening brand image (Sarkar, 2012). For example, Stella Artois partnered with water.org for the ‘Buy a Lady a Drink’ campaign, which aimed to increase access to clean drinking water through sales of branded glassware. Scoping and critique of alcohol industries’ green marketing practices is, therefore, another important avenue for future research. As consumers become increasingly concerned about the environment and are driven to make environmentally conscious purchases, industries will likely continue to implement measures aimed at offering ‘greener’ substitutes for traditional products (Sarkar, 2012). Although everyone is responsible for acting on climate change, young people’s general suspicion of industry greenwashing (Arnot *et al.*, 2023b) and their active role in lobbying for action (Arnot *et al.*, 2023a) underlie the importance of involving them in efforts to reduce alcohol’s climate and environmental effects.

### How climate change impacts alcohol use

There is substantial evidence demonstrating that climate change has both direct and indirect impacts on mental health (Lawrance *et al*., 2021; Ramadan and Ataallah, 2021). A survey of 10 000 young people aged 16–25 years, covering 10 different countries, found that 59% were ‘very’ or ‘extremely’ worried about climate change (Marks *et al*., 2021). Following motives literature, it is plausible to hypothesize that people may turn to alcohol to cope with climate anxiety and worries about the environment and the future [see (Vergunst *et al*., 2023) for an examination of plausible pathways]. Indeed, in a study of climate change and mental health among members of an Indigenous community in Canada, participants reported an enhanced likelihood that they would use alcohol and other drugs in response to changes in the environment and climate (Cunsolo Willox *et al*., 2013). Additionally, trauma relating to extreme weather events is associated with increased substance use (Beaudoin, 2011; Ramadan and Ataallah, 2021). The COVID-19 pandemic has also provided a natural experiment through which to understand changes in consumption in response to crises and suggests this relationship is far from straightforward, with variable patterns (both increases and decreases) found in response to uncertainty and new stresses (Acuff *et al*., 2022). However, continued monitoring of changes in consumption and demographic differences in these changes in response to new and ongoing social challenges is needed.

### Inequalities in generation of alcohol-associated emissions

The production of emissions from alcohol consumption also shows demographic and inter-country variations. Alcohol use is highest in the higher socio-demographic index countries and lowest in low- and middle-income countries [Socio-demographic index ‘is a summary measure of overall development, based on educational attainment, fertility, and income per capita within a location’ (Griswold *et al*., 2018, p. 1020).]. While consumption is greater in richer than poorer societies, there are more alcohol-related harms in lower-income societies [known as the alcohol harm paradox (Bellis *et al*., 2016; Boyd *et al*., 2022)], with some estimates suggesting that the rate of harm per litre in low-income countries is 3.7 times the rate in high-income countries (Room *et al*., 2022). Swedish estimates suggest that men’s alcohol consumption generates 90% higher GHG emissions than women’s, and that within the top 10% of consumers, GHG emissions were 202 kg of CO_2e_ for men and 134 kg of CO_2e_ for women, per person per year (Hallström *et al*., 2018). Higher rates of consumption for men in other countries, like the UK and Australia, mean there will likely be similar gender inequalities elsewhere too. Alcohol is a globalized commodity in high-income countries and consumption may occur a long way from production with, for example, Chilean wines consumed in Australia and Europe. Production and consumption of homebrew, often referred to as ‘unrecorded’ alcohol, are high in some low-income countries [for example, unrecorded alcohol is the most prevalent form consumed in Kenya (Mkuu *et al*., 2019)]. This form of alcohol tends to be produced and consumed locally, so the carbon generated is likely to be less. We echo calls for further investigations on how structural factors associated with production processes can exacerbate inequalities (McCarthy *et al*., 2023).

While climate change will jeopardize the health and well-being of the entire planet, people living in low- and middle-income countries, who contribute least to global emissions, are and will be disproportionately harmed (IPCC, 2023). The worst effects of climate change will be felt in the global south by people:

who are poor and undernourished, already ill, have insecure housing, farm degraded land, work in unsafe conditions, have little education, are deprived of their rights or live in places with poor health systems, limited resources and poor governance cannot influence decisions. (WHO, 2021)

Mitigating the environmental impacts is more challenging for those affected by social inequalities, who are less likely to have access to the required support and resources (Hadjikakou, 2017; Lawrance *et al*., 2021). Thus, climate change itself exacerbates social inequalities (Dominelli, 2011).

Reflecting that climate change is an issue that is produced, experienced and must also be acted on globally, Goal 13 of the United Nations’ SDGs is ‘Climate Action’ (United Nations, n.d.). Within this goal is a recognition that people and governments of wealthy global north countries, which produce the highest per capita proportions of emissions, to which alcohol constitutes a small albeit important contribution, have the greatest responsibility to act.

Alcohol policy is the responsibility of governments at various levels and should not be considered only within the jurisdiction of health departments. It requires global intersectoral and intragovernmental coordination to address commercial interests that profit from alcohol consumption (Buse and Hawkes, 2015; Room *et al*., 2022). Moreover, any efforts to reduce health harms from alcohol should consider alcohol’s impact on climate change and the environment, particularly given that changes in alcohol consumption driven by more environmentally conscious decisions will have concomitant benefits for individual health and society more broadly. Work by the WHO would be instrumental here, yet we note limited attention thus far on alcohol and the environment compared to tobacco, for which reports have considered negative environmental impacts [for example, (WHO, 2022a)]. Furthermore, there is very little mention of climate change or the environment in the alcohol action plan published in late 2022 (WHO, 2022b), although we note the WHO webinar on alcohol and the environment in September 2023 as part of their series ‘addressing blind spots to accelerate the implementation of effective policy interventions’ (WHO, 2023).

## CONCLUSION

Our planet is already facing the untold effects of climate change, and the time to act is quickly passing us by. Urgent systemic change is needed to mitigate further climate deterioration. While the most effective way to do this is to immediately cease fossil fuel extraction (IPCC, 2023), many commodities, including alcohol, have environmental costs. Concerns about climate change and other forms of environmental harm are more pervasive than ever, especially among younger generations. There is an unprecedented opportunity to implement upstream solutions to achieve environmental, and, in the case of alcohol, concomitant public health benefits. While alcohol is one of many fast-moving consumer goods with deleterious environmental effects, we propose generating a more systematic understanding of the climate harms and using this to advocate for change as a starting point for expanding to other products [e.g. (Pourchez *et al*., 2022)]. Given the projected impacts of human-caused climate change, our remit as public health researchers is to do what we can to understand these issues, educate others and seek solutions to them.

As part of this, researchers could meaningfully engage in other ways to explore how alcohol’s carbon footprint might be minimized. For example, those interested in the design and implementation of policies to improve health and prevent harm could examine the potential impact of policies regulating alcohol production and supply, which may lead to environmental gains while harnessing those concomitant benefits for health—if we can use the tax system to incentivize production of weaker-strength products, could it be used to incentivize more sustainable practice too? Individuals, particularly those in the top socio-economic quartile, have a responsibility to be aware of their contributions to emissions through their consumption, including alcohol products, and to consider ways of mitigating the environmental harms, perhaps through purchasing locally and sustainably produced products. As it stands, this knowledge is not readily and easily available to consumers. Previous research demonstrates that consumers need considerable assistance to make climate-friendly purchasing decisions (Thøgersen, 2021). Public health researchers already have a strong evidence base for educating the public on the harms from unhealthy commodities and motivating behaviour change around fast-moving consumer goods on which to draw here.

However, this is not a problem that is only addressed by taking individual responsibility for alcohol consumption or by producing new research. Governments and companies have a key responsibility to facilitate this, for example, by regulating alcohol companies and providing clearer information on the differential carbon footprints of alcohol products. The alcohol industry should not be allowed to evade responsibility and claim, through corporate social responsibility tactics, that they are doing enough. As the industry continues to reap billion-dollar profits (Jernigan and Ross, 2020), families, communities and nations are left to deal with the health and environmental consequences. Global corporations should work to reduce emissions and other causes of environmental harm resulting from the production and dissemination of their products, and governments must ensure they do this and be prepared to make the investments needed to facilitate the scale of systematic change needed (Friel *et al*., 2023).
